# Implantable Collamer Lens Versus Iris-Fixed Phakic Intraocular Lens Implantation to Correct Myopia: A Meta-Analysis

**DOI:** 10.1371/journal.pone.0104649

**Published:** 2014-08-12

**Authors:** Guan-Lu Liang, Jing Wu, Jun-Ting Shi, Jian Liu, Feng-Ying He, Wen Xu

**Affiliations:** 1 Eye Center, the 2nd Affiliated Hospital of Zhejiang University, College of Medicine, Hangzhou, Zhejiang, China; 2 Department of Ophthalmology, the 1st Affiliated Hospital of Zhejiang University, College of Medicine, Hangzhou, Zhejiang, China; University of Pennsylvania Perelman School of Medicine, United States of America

## Abstract

This study is a meta-analysis comparing the efficacy, predictability, and safety of correcting myopia via implantation of two types of phakic intraocular lens (PIOLs): the implantable collamer lens (ICL) and iris-fixed PIOL. The Cochrane library, Pubmed, and EMBASE were searched. Study selection, data exclusion, and quality assessment were performed by two independent observers. The pooled relative risk (RR), pooled standardized mean difference (SMD), and their 95% confidence intervals (CIs) were used to compare lenses. Seven studies, involving 511 eyes, were included. The pooled SMD in postoperative uncorrected distance visual acuity (UDVA) comparing ICLs to iris-fixed PIOLs was −0.22 (95% CI, −0.58 to 0.13; *P* = .22). The pooled RR values of UDVA of 20/20 or better and of 20/40 or better comparing ICLs to iris-fixed PIOLs were 1.15 (95% CI, 0.89 to 1.47; *P* = .29) and 1.01 (95% CI, 0.95 to 1.08; *P* = .75), respectively. The pooled RR of loss of best spectacle-corrected visual acuity (BSCVA) and gain in BSCVA comparing ICLs to iris-fixed PIOLs were 1.20 (95% CI, 0.24 to 6.00; *P* = .82) and 1.14 (95% CI, 0.89 to 1.48; *P* = .31), respectively. The pooled RR comparing ICLs to iris-fixed PIOLs was 0.78 (95% CI, 0.29 to 2.12; *P* = .63) for all reported complications and 2.80 (95% CI, 1.04 to 7.52; *P* = .04) for severe complications. The pooled RR of achieving a result within ±0.5 D (diopter) of the intended target comparing ICLs to iris-fixed PIOLs was 1.35 (95% CI, 1.04 to 1.77; *P* = .03). Overall, there is no significant difference in efficacy between the two types of PIOLs or in safety, except that the ICL is associated with a greater incidence of severe complications, especially anterior subcapsular cataract, primarily in the Version 2 and Version 3 groups. However, ICL has better predictability.

## Introduction

Refractive surgeries, including laser corneal refractive surgery, phakic intraocular lens (PIOL) implantation, and clear lens extraction (CLE) are among the approaches which have been developed to treat myopia and particularly high myopia. Although laser corneal refractive surgery is effective and safe in most cases [1], [2] has been the preferred option for refractive surgery for the past 20 years, physical limitations that are imposed by the thickness and curvature of cornea render it incapable of treating ametropic eyes in certain circumstances, especially those with high refractive errors [3]–[6]. Moreover, wound healing and biomechanical responses can occasionally lead to poor refractive predictability, prolonged visual recovery, instability of refraction, and loss of vision due to corneal irregularity and scarring [7].

Barsam et al. [8] reported that PIOL implantation is a safer method of correcting myopia in the range of −6.00 D to −20.00 D, effecting significantly less loss of best spectacle-corrected visual acuity (BSCVA) and better contrast sensitivity than corneal refractive surgery. PIOL implantation also scores higher on patient satisfaction and preference questionnaires. In recent years, with its reversibility, correction of a broader range of ametropia, preserved accommodation, faster visual recovery, more stable refraction, and better visual quality [9]–[11], PIOL implantation is gaining traction with more patients and refractive surgeons.

Generally, PIOLs are classified into three types, according to the position of IOL fixation: angle-supported anterior chamber (AC), iris-fixated AC, and posterior chamber (PC). Although there are many designs of angle-supported PIOLs, most have been withdrawn from the market due to safety concerns, particularly complications that are related to endothelial cell loss. Thus, fewer refractive surgeons consider angle-supported PIOLs as their primary option for correcting ametropia.

Iris-fixed AC PIOLs and implantable collamer lenses (ICLs) are approved by the US Food and Drug Administration (FDA) and have Conformity with European (CE) status. The Verisyse iris-fixed PIOL, marketed internationally as the Artisan iris-fixated PIOL by Ophtec and distributed in the US by Abbott Medical Optics, was the first PIOL to gain approval by the FDA in 2004, followed its foldable model, called the Artiflex/Veriflex. Dating back to the Worst-Fechner Claw Lens, iris-fixed PIOL is fixated to the peripheral iris with its two pincer-like haptics. Though iris-fixed PIOL has a long history, the currently available iris-claw model is basically the original IOL with few changes. Iris-fixated pIOLs have the advantages of “one size fits all” sizing, optimal distance from the crystalline lens and corneal endothelium. The Visian ICL, which is the most widely used sulcus-fixed PC PIOL (STAAR Surgical Company), gained FDA approval in December 2005 [7]. The ICL's designs and materials were refined in several clinical studies through a series of prototypes, first from the IC2020 and IC2020-M to ICM115, 120, 125, and 130; and then to the model V (version) 2, V3, and the current V4 Visian ICL.

The Visian ICL V4 addresses vaulting issues. This model has an additional 0.13- to 0.21-mm anterior vault due to the steeper radius of curvature of the base curve. The higher vault provides greater space between the posterior surface of the ICL and the anterior surface of the crystalline lens, allowing fluid changes in nutrients and preventing contact between the ICL and crystalline lens.

Although large clinical studies have demonstrated that the Visian ICL and Artisan/Verisyse PIOL have good efficacy, predictability, stability, and short-term safety [12]–[19], and despite several clinical comparative studies reporting disparate outcomes between the two types of PIOLs, no systematic review has compared their refractive outcomes. We performed a meta-analysis of all clinical comparative studies on the implantation of ICLs and iris-fixed PIOLs to compare their efficacy, predictability, and safety in correcting myopia to provide the most appropriate option for ophthalmologists and patients.

## Materials and Methods

### Search Strategy (Figure 1)

**Figure 1:**
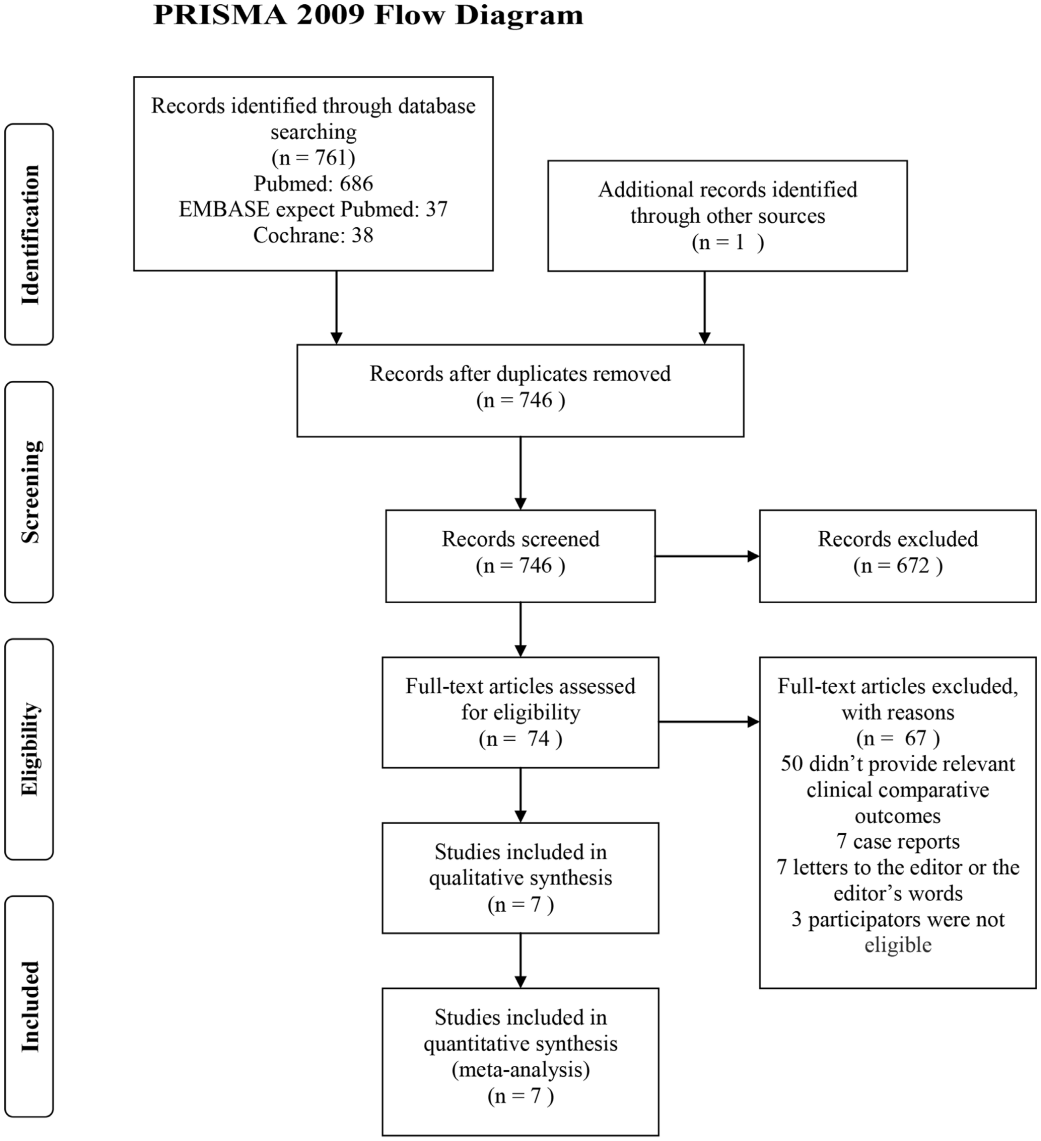
Flow chart of study selection process.

Literature in the Cochrane Library, including all subdatabases up to September 21, 2013; Pubmed from 1950 to September 21, 2013; and EMBASE from 1980 to September 20, 2013, were searched electronically using the terms “phakic intraocular lens” OR “posterior chamber phakic intraocular lens” OR “ICL” OR “implantable collamer lens” OR “implantable contact lens” OR “staar” OR “ciliary sulcus-fixed” OR “ciliary-fixed” OR “Artisan” OR “Verisyse” OR “Artiflex” OR “Veriflex” OR “iris-fixed” OR “iris-fixated” OR “iris-claw,” combined with “myopia” OR “shortsightedness” OR “nearsightedness.” The search was restricted to clinical studies and studies in EMBASE that were published in English. The titles and abstracts were scanned to exclude clearly irrelevant studies. The remaining full studies and their references were examined to determine whether they met the inclusion criteria.

### Study Selection

Two reviewers (G. Liang and J. Wu) performed the screen independently. The included studies had to: 1) compare ICLs (sulcus-fixed PIOLs) and iris-fixed PIOLs (Artisan/Verisyse or Artiflex/Veriflex PIOLs) with regard to correction of myopia, 2) follow up for a minimum of three months, and 3) report at least one of the outcomes below. When two studies were published by the same institution, the report with higher quality or the most recent publication was selected.

The following studies were excluded: 1) full texts that we were unable to obtain, even after contacting the authors, 2) studies in which the outcomes (discussed below) for these two PIOLs were not reported or impossible to calculate, 3) studies in which the subjects were aged younger than 18 years or had unstable refraction, 4) studies in patients who had preoperative ophthalmic diseases or contraindications such as keratoconus, cataract, glaucoma, retinal detachment, endothelial cell count less than 2000 mm^2^, and uveitis, or had undergone ophthalmic surgeries, especially refractive surgeries, and 5) studies that only reported complications, not refractive outcomes.

### Data Extraction

Information on the included studies—the name of the first author, the year of publication, the country in which the study was conducted, the number of eyes into which the PIOLs were implanted, the mean age of the patients who underwent PIOL implantation, the follow-up time, the preoperative spherical equivalent (SE) and uncorrected distance visual acuity (UDVA), and the refractive outcomes—were extracted independently by the two reviewers. Disagreements were resolved by discussion with a senior reviewer (W. Xu).

The extracted refractive outcomes were 1) efficacy: the mean postoperative UDVA (log MAR), the number of the eyes that achieved a UDVA of 20/20 or better, or the number of the eyes that achieved a UDVA of 20/40 or better postoperatively; 2) safety: the number of the eyes that lost or gained one or more lines (Snellen visual chart) of postoperative BSCVA and the number of complications in the studies; and 3) predictability: the number of the eyes that achieved a result within ±0.5 D of the intended target. If data were unavailable or could not be derived from the publication, we attempted to acquire additional information by contacting the corresponding authors by email.

### Qualitative Assessment

Because randomized controlled trials (RCTs) and non-RCTs were included in this meta-analysis, the Downs and Black quality method, which is appropriate for RCTs and non-RCTs, was used to assess the qualities of the studies [20]. The Downs and Black Scale comprises 27 criteria that evaluate the reporting, external validity, internal validity, selection biases, and power of the studies. Based on quality score, each study is grouped into one of four levels: 26-28, 20–25, 15–19, and ≤14 [21]. Higher scores indicate higher quality. Because few studies reported the study's power, this parameter was omitted. Thus, the quality of each study was considered excellent (21–23), good (15–20), fair (10–14), and poor (≤9) by the two independent reviewers, and disagreements were resolved through discussion with a third reviewer (W. Xu).

### Statistical analysis

The data of both ICLs and iris-fixed PIOLs on efficacy, safety and predictability—the number of the eyes that achieved a UDVA of 20/20 or better and a UDVA of 20/40 or better postoperatively, the number of the eyes that lost or gained one or more lines of postoperative BSCVA, the number of complications, and the number of the eyes that achieved a result within ±0.5 D of the intended target—were aggregated into 2×2 tables, and the pooled RRs and 95% CIs comparing ICLs to iris-fixed PIOLs implantation were performed.

The estimated RR comparing ICLs to iris-fixed PIOLs was considered statistically significant at *P*<0.05 if the 95% CI did not include the value “1”. The data on efficacy—i.e., the mean postoperative UDVA (log MAR)—were used to calculate the SMD comparing ICLs to iris-fixed PIOLs, which was considered statistically significant at P<0.05 if the 95% CI did not include the value “0”.

The chi-square and I^2^ tests were used to assess the heterogeneity of the studies [22], [23]. In our study, the random-effects model, which calculates a more conservative value and is less influenced by the weight of each study [24], [25], also weights the studies relatively more equally than a fixed-effect analysis in the presence of heterogeneity as per the Cochrane Collaboration handbook, was used, regardless of heterogeneity. In surgical research, a meta-analysis that uses the random-effects model is preferable, because patients from the clinical centers have varying risk profiles and selection criteria. The seven studies in our meta-analysis differed in design, which was a potential source of heterogeneity between studies.

Publication bias was assessed by Egger regression asymmetry test [26] and Begg adjusted rank correlation test [27]. Because a subgroup analysis is not appropriate for studies with small sample sizes, sensitivity analyses were conducted by omitting the data from a study that was believed to cause heterogeneity and effect misleading conclusions. Moreover, sensitivity analyses were performed to verify that the conclusions were robust.

Yates's correction was used for studies that contained a value of “0” in one cell for the number of events of interest in one of the two groups [28], [29]. These “zero cells” created problems in computing the ratio and standard error of the treatment effect. This issue was resolved by adding a value of 0.5 to each cell of the 2×2 table for the study in question, which was done automatically in Stata. If there were no events for the ICL and iris-fixed PIOL groups, the study was discarded. The statistical software program Stata/SE 12.0 was used for these analyses (Stata Corporation, College Station, TX).

To translate the results on the complications into benefits of the clinical outcomes, the following values were calculated: 1) absolute risk reduction (ARR), the difference in the incidence of all reported postoperative complications between the ICL and iris-fixed PIOL groups; 2) the number of patients needed to treat (NNT) using ICL to achieve one additional favorable outcome (NNT = 1/ARR); 3) absolute risk increase (ARI), the difference in the incidence of severe postoperative complications between the two groups; and 4) NNH, the number of patients needed to treat to harm one more patient in the therapy (NNH = 1/ARI).

## Results

### Study Characteristics

A total of 762 references, comprising 761 from the database search and one additional reference from other sources, were identified—16 were duplicate studies, and 672 were not relevant on review of the title and abstract. Ultimately, 74 references remained and were assessed by examining the entire text carefully. Of the 74 references, 50 were reviews, studies without clinical comparative data, or studies that reported irrelevant comparisons and outcomes; seven were case reports; seven were letters to the editor or the editor's remarks; and three included subjects aged younger than 18 years.

Thus, a total of seven references [30]–[36] met our inclusion criteria and were included in our study. These studies comprised two RCTs [30], [35], one prospective nonrandomized comparative study [34], and four cohort studies [31]–[33], [36], involving a total of 511 eyes, of which 190 (37.18%) underwent ICL implantation and 321 (62.82%) underwent iris-fixed PIOL implantation. The characteristics of the included studies are presented in Table 1.

**Table 1:** Characteristics of 7 included studies.

Studies	Country	Number	Age	Follow-up	Pre-SE^a^(Diopter)	Pre-UDVA^b^
(Author Year)		(ICL/Iris-fixed)	(ICL/Iris-fixed)	(ICL/Iris-fixed)	(ICL/Iris-fixed)	(ICL/Iris-fixed)
Awadein [30]	Egypt	24/24	NA^c^	6M^d^	−11.48±2.04/	1.48±0.18/
2013					−10.97±1.91	1.42±0.23
Wachler [31]	USA	30/31	39.6/40.9	3M	−11.48±3.84/	2.00±0/
2009					−12.31±3.50	1.87±0.28
Hassaballa [32]	Egypt	26/42	29.85/28.25	1D^e^,1W^f^,1M	−12.44±4.15/	NA/
2011				3M,6M,1Y^g^	−12.89±3.78	NA
Lee [33]	Korea	30/31	28.6/29.0	1M,3M,6M	−9.80±2.49/	1.67±0.33/
2011					−10.04±3.48	1.65±0.52
Menezo [34]	Spain	21/137	31.3/36.2	18M	−16.00±5.05/	1.52±0.55/
2004					−16.17±2.75	1.85±0.40
Ghoreishi [35]	Iran	20/20	27/27	1Y	NA/	1.69±0.17/
2011					NA	1.67±0.17
Zhang [36]	China	39/36	28.2/31.9	3M/1Y	−14.29±3.77/	NA/
2011					−15.44±3.70	NA

a Spherical Equivalent;

b Uncorrected Distance Visual Acuity;

c data Not Available;

d Month;

e Day;

f Week;

g Year.

Table 2 summarizes the quality of the seven studies. Their quality scores ranged from 12 to 20, with an average of 16.14. Based on the Quality Assessment Score (QAS), five studies [30]–[33], [35] were rated good and two [34], [36] were rated fair.

**Table 2:** Scores of Downs and Black Scale.

Studies	Reporting	External	Internal	Internal	Total
(Author)		Validity	Validity Bias	Validity Confounding	Scores
Awadein [30]	9	3	4	4	20
Wachler [31]	10	1	4	2	17
Hassaballa [32]	9	1	4	2	16
Lee [33]	9	1	4	2	16
Menezo [34]	8	1	3	1	13
Ghoreishi [35]	9	2	4	4	19
Zhang [36]	7	1	3	1	12

### Efficacy

**Figure 2:**
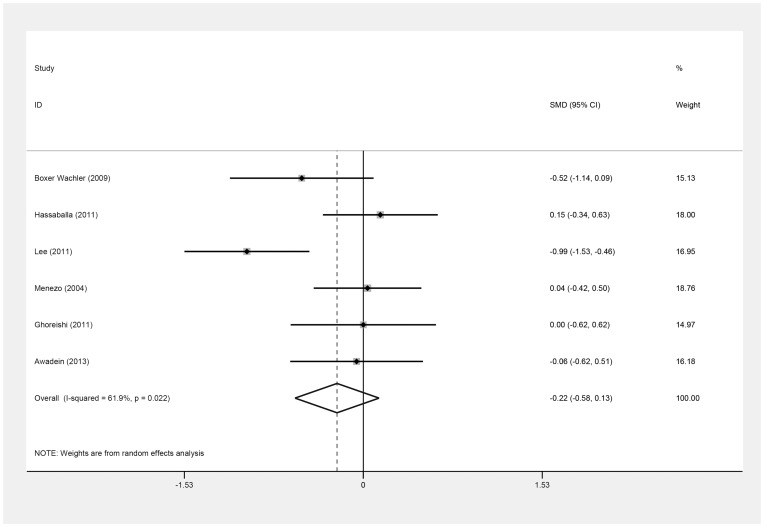
Forest plot comparing uncorrected distance visual acuity (log MAR) after implanting Implantable Collamer Lens and iris-fixed phakic intraocular lens (First author and year of publication of each study given) (CI =  confidence interval; SMD =  standardized mean difference).

**Figure 3:**
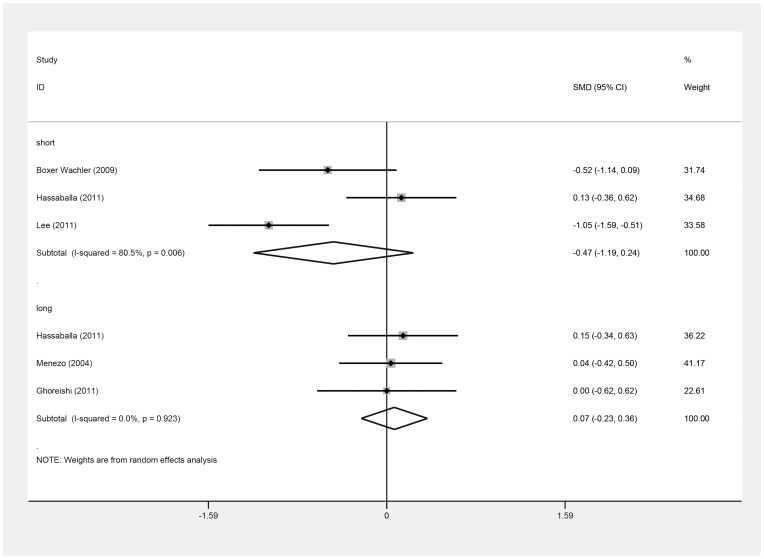
Forest plot comparing uncorrected distance visual acuity (log MAR) after implanting Implantable Collamer Lens and iris-fixed phakic intraocular lens according to different following-up time. Short term means short-term following-up, long term means long-term following-up. (First author and year of publication of each study given) (CI =  confidence interval; SMD =  standardized mean difference).

Six of the seven studies provided data [30]–[35] for calculating the SMD of postoperative UDVA (log MAR). Table 3 shows the clinical data on the log MAR values of the six studies [30]–[35]. There was no difference in postoperative UDVA (log MAR) between the ICL and iris-fixed PIOL groups (SMD = −0.22; 95% CI, −0.58 to 0.13, *P* = .22; I^2^ = 61.9%) (Figure 2). Omitting Lee's study [33], similar results were obtained (SMD = −0.05, 95% CI, −0.29 to 0.19; *P* = .71; I^2^ = 0%). In all these seven studies, three [31]–[33] reported the postoperative UDVA at the three-month follow-up, and another three [32], [34], [35] reported postoperative UDVA after greater than one year of follow-up. Their results did not differ significantly between groups at the short-term (SMD = −0.47; 95% CI, −1.19 to 0.24; *P* = .19; I^2^ = 80.5%) or long-term follow-up (SMD = 0.07; 95% CI, −0.23 to 0.36; *P* = .65; I^2^ = 0.0%) (Figure 3). There was no evidence of publication bias (Begg's test, *P* = .71; Egger's test, *P* = .56).

**Table 3:** Postoperative uncorrected distance visual acuity (log MAR).

Studies	ICL	Iris-fixed	Follow-up
(Author)	(Mean±SD)	(Mean±SD)	
Awadein [30]	0.21±0.17	0.22±0.18	6 month(last)^a^
Wachler [31]	0.02±0.08	0.09±0.18	3 month(last)
Hassaballa [32]	0.41±0.16	0.39±0.15	3 month
	0.41±0.15	0.39±0.13	1 year(last)
Lee [33]	−0.04±0.09	0.06±0.10	3 month
	−0.04±0.08	0.03±0.06	6 month(last)
Menezo [34]	0.30±0.17	0.29±0.29	18 month(last)
Ghoreishi [35]	0.04±0.06	0.04±0.05	1 year(last)

a at the last follow-up visit.

**Figure 4:**
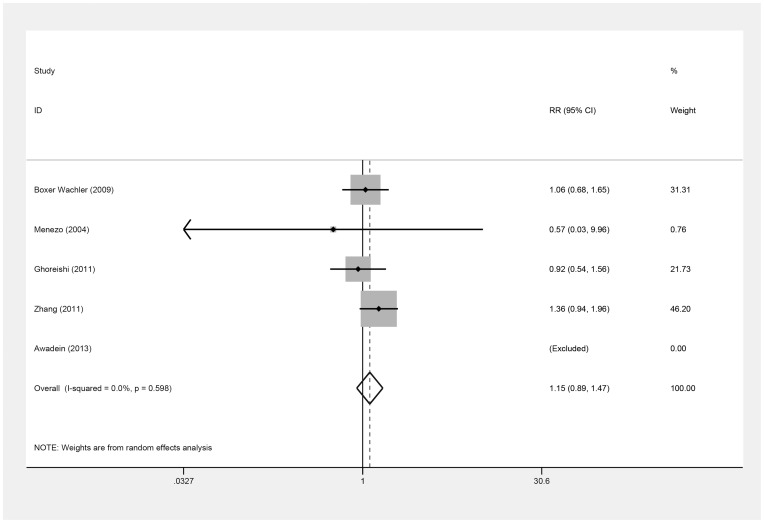
Forest plot comparing the number of eyes achieving 20/20 (Snellen) or better after implanting Implantable Collamer Lens and iris-fixed phakic intraocular lens. (First author and year of publication of each study given) (CI =  confidence interval; RR =  relative risk).

**Figure 5:**
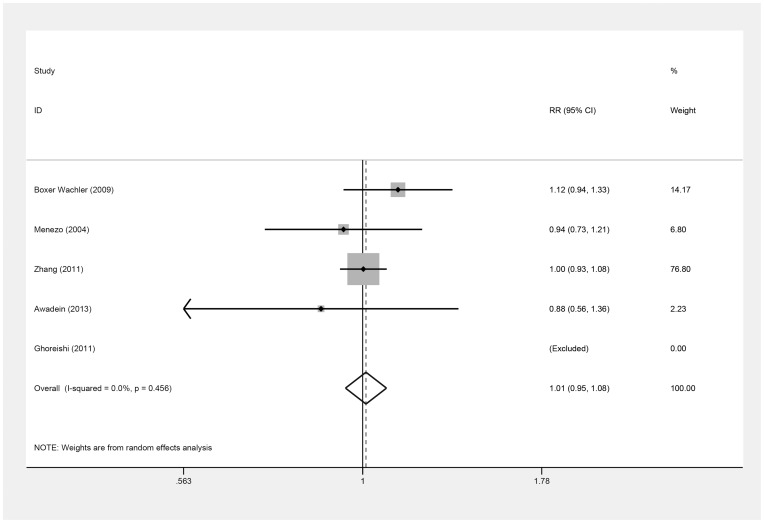
Forest plot comparing the number of eyes achieving 20/40 (Snellen) or better after implanting Implantable Collamer Lens and iris-fixed phakic intraocular lens. (First author and year of publication of each study given) (CI =  confidence interval; RR =  relative risk).

The number of the eyes that achieved a UDVA of 20/20 or better and a UDVA of 20/40 or better postoperatively were also used to estimate the efficacy (Table 4, Table 5) in five studies [30], [31], [34]–[36], but these values did not differ between the ICL and iris-fixed PIOL groups [(RR = 1.15; 95% CI, 0.89 to 1.47, *P* = .29; I^2^ = 0.0%) (Figure 4) (RR = 1.01; 95% CI, 0.95 to 1.08, *P* = .75; I^2^ = 0.0%) (Figure 5), respectively]. On omitting Zhang's study [36], the results remained similar [(RR = 0.99; 95% CI, 0.70 to 1.39, *P* = .95; I^2^ = 0.0%) (RR = 1.02; 95% CI, 0.86 to 1.21, *P* = .79; I^2^ = 24.4%), respectively]. Overall, there was no evidence of publication bias (Begg's test, *P* = .31; Egger's test, *P* = .39) (Begg's test, *P* = .31; Egger's test, *P* = .83).

**Table 4:** The number of eyes that achieved uncorrected distance visual acuity of 20/20 or better at the last follow-up visit.

Studies	ICL (Number)		Iris-fixed (Number)	
(Author)	Achieve	Not-achieve	Achieve	Not-achieve
Awadein [30]	0	24	0	24
Wachler [31]	16	8	12	7
Menezo [34]	0	21	5	132
Ghoreishi [35]	11	9	12	8
Zhang [36]	28	11	19	17

**Table 5:** The number of eyes that achieved uncorrected distance visual acuity of 20/40 or better at the last follow-up visit.

Studies	ICL (Number)		Iris-fixed (Number)	
(Author)	Achieve	Not-achieve	Achieve	Not-achieve
Awadein [30]	14	10	16	8
Wachler [31]	24	0	17	2
Menezo [34]	16	5	111	26
Ghoreishi [35]	20	0	20	0
Zhang [36]	38	1	35	1

### Safety

**Figure 6:**
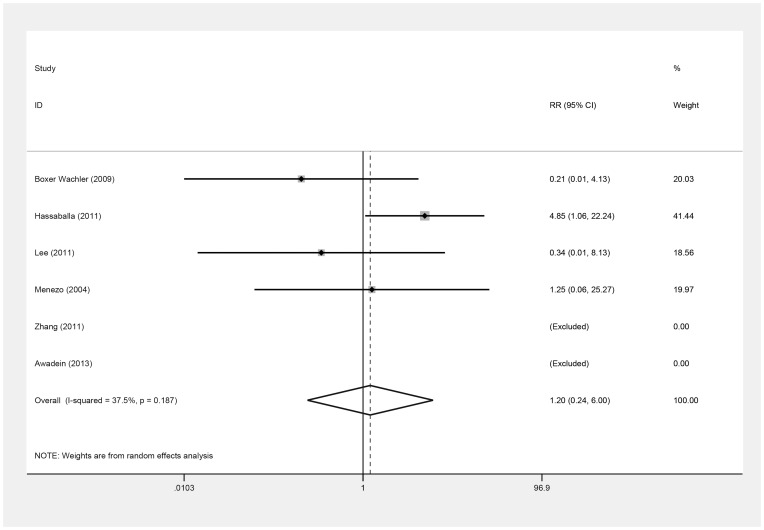
Forest plot comparing the number of eyes losing one or more lines of best spectacle corrected visual acuity after implanting Implantable Collamer Lens and iris-fixed phakic intraocular lens. (First author and year of publication of each study given) (CI =  confidence interval; RR =  relative risk).

Six studies [30]–[34], [36] assessed safety, based on the number of eyes that lost one or more lines of postoperative BSCVA (Table 6). No significant difference was observed between groups (RR = 1.20; 95% CI, 0.24 to 6.00; *P* = .82; I^2^ = 37.5%) (Figure 6). Omitting Lee et al. [33], this result was similar (RR = 1.53; 95% CI, 0.23 to 10.12; *P* = .66; I^2^ = 45.1%). By Begg's and Egger's tests, there was no evidence of substantial publication bias (*P* = .73, *P* = .08).

**Table 6:** The number of the eyes losing one or more lines of best spectacle corrected visual acuity at the last follow-up visit.

Studies	ICL (Number)		Iris-fixed (Number)	
(Author)	Loss	Not-loss	Loss	Not-loss
Awadein [30]	0	24	0	24
Wachler [31]	0	30	2	29
Hassaballa [32]	6	20	2	40
Lee [33]	0	30	1	31
Menezo [34]	0	21	2	135
Zhang [36]	0	39	0	36

**Figure 7:**
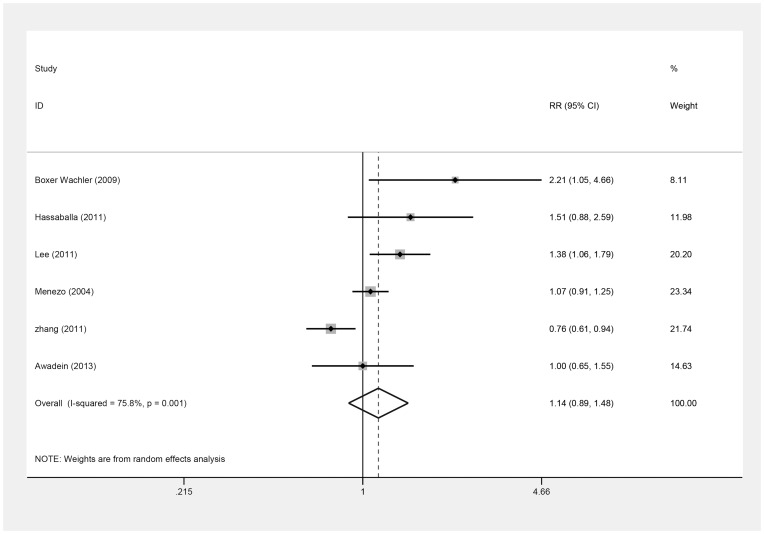
Forest plot comparing the number of eyes gaining one or more lines of spectacle corrected visual acuity after implanting Implantable Collamer Lens and iris-fixed phakic intraocular lens. (First author and year of publication of each study given) (CI =  confidence interval; RR =  relative risk).

These six studies [30]–[34], [36] also reported the number of the eyes that gained one or more lines of postoperative BSCVA (Table 7), which did not differ (RR = 1.14; 95% CI, 0.89 to 1.48; *P* = .31; I^2^ = 75.8%) (Figure 7); this result remained the same on omitting Zhang's study [33], [36] (RR = 1.26; 95% CI, 0.99 to 1.60, *P* = .06; I^2^ = 56.8%). There was no publication bias by Begg's and Egger's tests (*P* = .45, *P* = .32).

**Table 7:** The number of the eyes gaining one or more lines of best spectacle corrected visual acuity at the last follow-up visit.

Studies	ICL (Number)		Iris-fixed (Number)	
(Author)	Gain	Not-gain	Gain	Not-gain
Awadein [30]	15	9	15	9
Wachler [31]	15	15	7	24
Hassaballa [32]	14	12	15	27
Lee [33]	28	2	21	10
Menezo [34]	19	2	116	21
Zhang [36]	28	11	34	2

**Figure 8:**
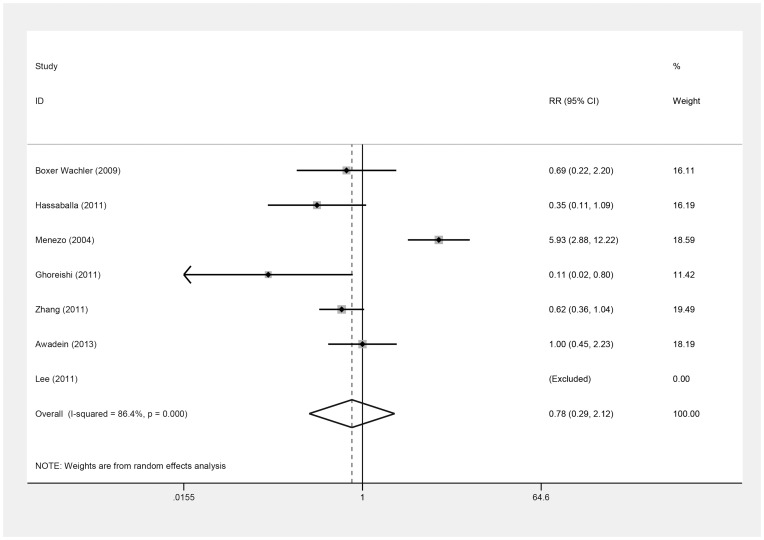
Forest plot comparing all the reported complications of eyes after implanting Implantable Collamer Lens and iris-fixed phakic intraocular lens. (First author and year of publication of each study given) (CI =  confidence interval; RR =  relative risk).

All seven studies [30]–[36] provided information on complications (Table 8). The incidence of the complications was also assessed, based on the safety index of the two types of PIOLs. The incidence of all complications did not differ significantly between groups (RR = 0.78; 95% CI, 0.29 to 2.12; *P* = .63; I^2^ = 86.4%) (Figure 8). By omitting Menezo's study [34], in which the V2 and V3 ICLs and their replacement, the V4 ICL [37], [38], were implanted, while other included studies didn't implant the former V2 and V3 ICL which were reported to result in obviously more complications because of the design, the ICLs appeared to have fewer complications (RR = 0.59; 95% CI, 0.36 to 0.97; *P* = .04; I^2^ = 29.0%). Begg's and Egger's tests indicated no evidence of substantial publication bias (*P* = .71, *P* = .56).

**Table 8:** Descriptions of all complications in 7 included studies.

Studies	Lens	Complications	Management	Outcomes	Follow-up
Awadein [30]	ICL	8 night vision symptom	NAa	NA	6Mb
	Iris-fixed	8 night vision symptom	NA	NA	6M
Wachler [31]	ICL	2 subcapsular opacity	NA	NA	3M
		1 halos	NA	NA	
		1vault absence	IOL^c^ Exchange	UDVA^d^:20/25	
	Iris-fixed	5 foreign body sensation	NA	NA	3M
		1 significant dry eye	NA	NA	
Hassa-	ICL	1 angle closure glaucoma	IOL Exchange	IOP^e^ normal	1Y^f^
Balla [32]		2 pigment dispersion	NA	NA	
	Iris-fixed	1 malignant glaucoma	Anterior vitrectomy	IOP normal	1Y
		12 pigment dispersion	NA	NA	
		1 displacement	reenclavation	safe	
Lee [33]	ICL	none	none	none	6M
	Iris-fixed	none	none	none	6M
Menezo [34]	ICL	8 pigment deposits	NA	NA	18M
		2 subcapsular cataract	NA	NA	
	Iris-fixed	9 pigment deposits	NA	NA	18M
		2 nuclear cataract	NA	NA	
Ghore-	ICL	1 subcapsular cataract	NA	NA	1Y
Ishi [35]	Iris-fixed	1 subcapsular cataract	NA	NA	1Y
		8 trace cells and flare	NA	NA	
Zhang [36]	ICL	5 high IOP	drugs treatment	all IOP normal	1Y
		5 lens opacity	NA	NA	
		2 halos	NA	NA	
	Iris-fixed	8 high IOP	drugs treatment	all IOP normal	1Y
		1 lens opacity	NA	NA	
		3 halos	NA	NA	
		36 pigment dispersion	NA	NA	

a data Not Avaliable;

b Month;

c Intraocular Lens;

d Uncorrected Distance Visual Acuity;

e Intraocular Pressure;

f Year.

Next, we examined whether the severe complications adversely affected the patients. We defined the severe complications as follows: 1) complications that needed long-term clinical intervention or surgery, such as lens opacities; cataracts; high intraocular pressure (IOP) that could not be controlled by short-term drugs; glaucoma; retinal detachment; obvious loss of corneal endothelial cells that led to corneal edema or other clinical symptoms; and severe uveitis that had anterior chamber cells, flare, pain, ciliary hyperemia, keratic precipitates, or other signs of uveitis; and 2) any reasons that led to IOL exchange or removal.

**Figure 9:**
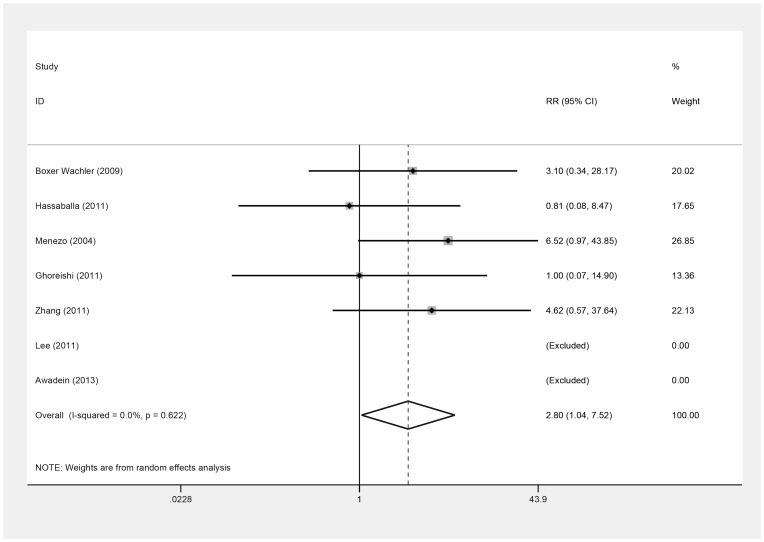
Forest plot comparing the eyes with severe complications after implanting Implantable Collamer Lens and iris-fixed phakic intraocular lens. (First author and year of publication of each study given) (CI =  confidence interval; RR =  relative risk).

There was a greater incidence of severe complications in the ICL group versus the iris-fixed PIOL group (RR = 2.80; 95% CI, 1.04 to 7.52; *P* = .04; I^2^ = 0.0%) (Figure 9). Similarly, we omitted Menezo's study [34] to do the sensitivity analysis. The V2, V3, and V4 ICLs that were all implanted in this study, increasing the incidence of postoperative lens opacity, even cataract because of the small vault of V2, V3 ICLs. V3 ICLs were associated with a 9.2% incidence of cataract versus 0.8% for V4 ICLs (*P*<.001), according to the FDA clinical trial [39], we found no significant difference in severe complications between the groups (RR = 2.06; 95% CI, 0.65 to 6.52; *P* = .22; I^2^ = 0.0%). Overall, there was no evidence of publication bias (Begg's test, *P* = .09; Egger's test, *P* = .05).

The incidence of all reported complications was 20.0% (38 of 190) in the ICL group compared with 26.2% (84 of 321) in the iris-fixed PIOL group. The ARR was 0.062, which translated into an NNT of 16. However, the incidence of severe complications was 6.32% (12 of 190) in the ICL group, significantly higher than the 2.18% rate (7 of 321) in the iris-fixed PIOL group. The ARI and NNH values were 0.041 and 24, respectively.

### Predictability

**Figure 10:**
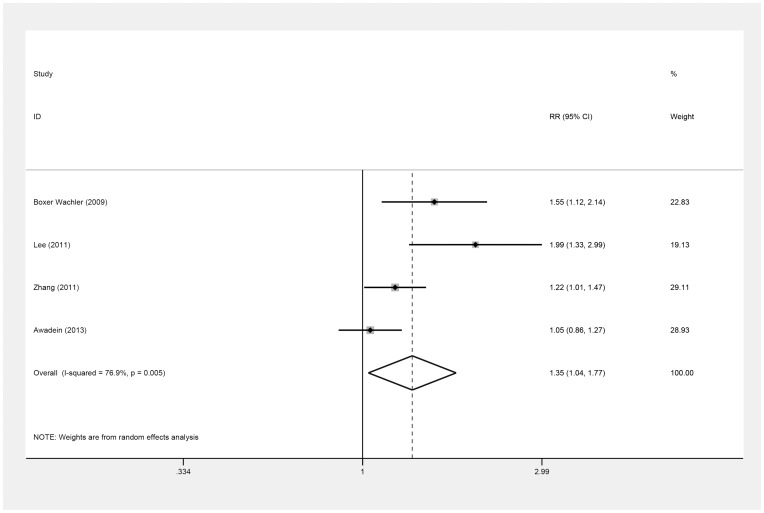
Forest plot comparing the number of eyes achieving a postoperative spherical equivalent within ±0.5 D of the intended target after implanting Implantable Collamer Lens and iris-fixed phakic intraocular lens. (First author and year of publication of each study given) (CI =  confidence interval; RR =  relative risk).

Four studies [30], [31], [33], [36] provided data on the number of eyes that achieved a postoperative SE within ±0.5 D of the intended target (Table 9), indicating that predictability in the ICL group was better than in the iris-fixed PIOL group (RR = 1.35; 95% CI, 1.04 to 1.77; *P* = .03; I^2^ = 76.9%) (Figure 10). We did not perform a sensitivity analysis due to the small number of studies. Overall, there was no evidence of publication bias (Begg's test, *P* = .31, Egger's test, *P* = .07).

**Table 9:** The number of eyes achieving a postoperative spherical equivalent within ±0.5 D of the intended target.

Studies	ICL (Number)		Iris-fixed (Number)	
(Author)	Achieve	Not-achieve	Achieve	Not-achieve
Awadein [30]	22	2	21	3
Wachler [31]	27	3	18	13
Lee [33]	27	3	14	17
Zhang [36]	37	2	28	8

## Discussion

Several clinical studies [17], [40] and a review [41] have reported satisfactory refractive outcomes with ICL and iris-fixed PIOL implantation for correcting myopia. Although some studies compared refractive outcomes between these PIOLs, the conclusions differed, and no review has performed a systematic comparison of ICL and iris-fixed PIOL implantation for myopia. Thus, we performed a meta-analysis to compare the efficacy, safety, and predictability of the two types of PIOLs to guide the selection of PIOLs in correcting myopia.

In the seven studies [30]–[36], there was no significant difference in efficacy or safety, except for the incidence of severe complications, between the ICL and iris-fixed PIOL groups for correction of high myopia, and ICL implantation had better predictability. However, these findings should be examined cautiously, because the number of the studies was limited and the study types differed. Based on NNT values, postoperative complications were more likely to be limited with ICL. An NNT value of 16 for all reported complications suggests that 16 patients on average would require ICL implantation to reduce one certain complication than iris-fixed PIOL implantation. In contrast, with regard to severe postoperative complications, the NNH value was as high as 24, indicating that 24 patients on average could be treated to increase one certain severe complication with ICL implantation than iris-fixed PIOL

As is known to us, there are several versions of ICLs. Among the seven included studies, five study [30]–[32], [35]–[36] implanted the V4 ICL, Lee's study [33] included toric ICL which added a cylinder on an axis specific of the optical zone on the base of the V4 ICL, and the haptic design of the toric ICL is identical to that of a V4 ICL in terms of size, thickness, and configuration. Menezo's study [34] implant not only the V4 ICL but also the obsolete V2 and V3 ICL which cause more complications because of the design of the smaller vault. To follow meta-analysis's principle, we included all the data conforming to the inclusion criteria to reveal the outcomes objectively. What's more, we did sensitivity analysis to analyze the heterogeneity and the robustness of the conclusion.

The most significant parameter of the efficacy of refractive surgery is visual acuity. However, the efficacy was not standardized between studies, and disparate cutoffs in postoperative UDVA were used. We chose the outcomes that are used most frequently in the included studies. If only the mean and standard deviation of log MAR values were selected, the outlines of the results would go into hiding [42]. Thus, we combined the mean log MAR of UDVA with the number of eyes with a visual acuity of 20/20 or better and the number of the eyes with a visual acuity of 20/40 or better to compare the efficacy between PIOLs to reflect the efficacy more clear and comprehensive.

There was no difference in efficacy between groups, based on the three indicators. With regard to log MAR values of UDVA, we omitted a study by Lee et al. [33] to determine whether the result was robust, in which the patients received toric ICLs or toric iris-fixed PIOLs with a different IOL design from other included studies called “toric” that could correct both astigmatism and myopia, possibly leading to an incorrect conclusion. Excluding this study, associations were strengthened. We also analyzed the log MAR values of UDVA at various follow-up times and obtained consistent results.

We performed a sensitivity analysis of the number of eyes with visual acuity of 20/20 or better and 20/40 or better by omitting Zhang's study [36], which had the lowest QAS score but the highest weight, which might have underestimated or overestimated the result.

All results consistently demonstrated the robustness of the conclusion. Only Boxer's study [31] and Lee's study [33] showed a significant difference in postoperative UDVA, Boxer's study [31] reported similar monocular UDVA but better binocular acuity in the ICL group (*P* = .007), attributing this difference to two reasons: significant induced astigmatism that accounted for the large incision in the Artisan PIOL group and the position of the PIOLs—the iris-fixed PIOL was located on the iris plane, and the ICL lay on the ciliary sulcus plane, which was closer to the nodal point of the eye and might have resulted in better outcomes.

Lee's study [33] showed no significant difference in log MAR BSCVA but had better outcomes with the ICL in terms of log MAR UDVA. Lee et al. opined that residual astigmatism might have influenced the significant differences in log MAR UDVA. Like Boxer, Lee et al. attributed the better outcome with the ICL to the smaller incision length, based on an analysis of manifest cylinder power and astigmatism vector. It is possible that these factors influenced the results, although their effects were not sufficient to reach statistical significance between groups. The heterogeneity of the six studies [30]–[35] that reported log MAR values of UDVA was 61.9%, and after omitting Lee's study [33], the I^2^ for heterogeneity declined to 0.0%, suggesting that small differences in intervention design cause high heterogeneity.

There were two studies [33], [36] that also reported efficacy indexes (mean postoperative UDVA and mean preoperative BSCVA) of 1.155 and 1.009 for the ICL group and 1.06 and 1.15 for the iris-fixed group, respectively. The results indicated good efficacy of the ICL and iris-fixed PIOLs for myopia. Ghoreishi et al. [35] and Awadein et al. [30] recorded similar postoperative contrast sensitivities at 3, 6, 12, and 18 cycles/degrees and obtained similar outcomes between groups between groups.

Usually, safety outcomes in refractive surgery are expressed as a loss or gain in Snellen lines of postoperative BSCVA. In this study, we also added the incidence of complications as an index of safety outcomes. There was no difference in the loss or gain of one or more lines of BSCVA postoperatively. The loss of postoperative BSCVA remained robust after omitting Lee's study [33] which implanted toric ICL and toric iris-fixed PIOLs and are designed to correct both astigmatism and myopia; the gain in postoperative BSCVA was also robust after omitting Zhang's study [36] which received the lowest QAS.

We observed a greater, albeit insignificant incidence of all referred complications with iris-fixed PIOLs and a significantly higher incidence of severe complications in the ICL group. Chen et al. [43] analyzed three types of PIOLs: angle-supported AC, iris-fixed, and PC PIOLs. After including all relevant studies, involving more than 2000 eyes, their results were similar with ours, reporting 11 and seven types of adverse events with an incidence that exceeded 1% in the iris-fixed PIOL and PC-PIOL group, respectively. Because the iris-fixed PIOLs were fixed on the iris by haptics, there were many complications that were related to the iris and pupils, such as iris atrophy, pigment deposits, pigment dispersion, pupil ovalization, and halos/glare, as in Zhang et al. [36], nearly all eyes appeared to have greater or less pigment dispersion, although it did not cause any severe harm, according to the follow-up. Cataract was the most common cause of PIOL reimplantation. In Chen et al. study, the incidence of cataracts in the ICL group was 8.48%, higher than 1.11% in the iris-fixed PIOLs group [43].

On omitting the study that implanted several versions of the ICLs, especially V2 and V3 with smaller vaults that might have led to statistical bias and have been eliminated from the market, while other included studies didn't implant these two versions of ICLs, there was no significant difference in the incidence of severe complications between groups, but the incidence of all referred complications was higher in the iris-fixed PIOL group. These changes demonstrate that the conclusions regarding the complications were not robust and should be interpreted with caution. These changes could be explained by the following reasons: 1) no standardized definition of the complications or adverse events; 2) no sufficient follow-up time to observe adverse events and evaluate safety adequately; and 3) the limited sample size of subjects with complications. There was significant heterogeneity among studies that reported all complications, but on omitting Menezo et al., the heterogeneity fell to 29.0%.

As discussed, implanting V2 and V3 ICLs could have led to a higher incidence of cataracts. In an FDA trial, the incidence of asymptomatic anterior subcapsular opacities was 12.6% in the V3 group and 2.9% in the V4 group (*P*<.001). The rate of clinically significant cataract was 9.2% in the V3 group and 0.8% in the V4 group (*P*<.001) due to the poor vault in the former [44]. Thus, the design of a PIOL significantly influences heterogeneity.

We also attempted to analyze the effects of patient age, sample size, anterior chamber depth (ACD), race, the definition of complications, the degree of myopia, and study design on heterogeneity. However, due to limited number of studies, we were unable to to assess their influence on heterogeneity.

The safety index—the ratio of mean postoperative UDVA to mean preoperative UDVA—was 1.2 and 1.31 in the ICL and iris-fixed PIOL groups in Zhang et al. [36], 1.278 and 1.187 in Lee's study [33], and 1.18 and 1.02 in Hassaballa's study [32], respectively. Three studies [30], [35], [36] observed a high-profile loss in the number of endothelial cells after PIOL implantation. Awadein et al. [30] reported that although endothelial loss was higher with the iris-fixed PIOL than the ICL, the difference was not significant (*P* = .11). Ghoreishi et al. [35] also found no significant difference in postoperative endothelial cell count (*P* = .72) and coefficient of variation (*P* = .25) between PIOLs. Zhang et al. [36] performed a vertical comparison of preoperative and postoperative endothelial cell count of the PIOLs and concluded that the difference did not reach statistical significance. All studies reported good safety with the ICL and iris-fixed PIOLs.

With regard to predictability, ICLs had better predictability, based on the number of eyes the achieved a postoperative SE within ±0.5 D of the intended target. We did not perform a sensitivity analysis of the robustness of the results, because there were too few studies.

ICL implantation was relatively faster and required less technique than iris-fixed PIOL implantation [31]. In addition, Lee et al. [33] reported that ICL patients experienced faster recovery times and greater operative stability compared with iris-fixed patients. However, selecting ICL length based on ACD, white-to-white (WTW), or sulcus-to-sulcus (STS) measurements was important but difficult. Improper ICL sizes can result in dissatisfactory refractive outcomes and several common and severe complications [39], [45]. Iris-fixed (Artisan/Verisyse) PIOL implantation is a longer procedure and requires incision construction, enclavation, and incision suturing. However, its “one size fits all” property prevented complications from sizing errors [46], giving it an advantage over the Visian ICL.

Our meta-analysis had some limitations. First, unpublished articles were not included, which could effect publication bias, although no significant evidence of publication bias was observed by Egger's and Begg's test. Second, the studies had a small sample size, insufficient follow-up duration, and several designs, although including as many studies as possible is encouraged to prevent misleading conclusions from being drawn if the studies are homogeneous [47]. We adopted a random-effects model and performed a sensitivity analysis to verify the robustness of the results, except for the complications which was referred above. Third, the effects of age, degree of myopia, preoperative BSCVA, and other factors were not analyzed due to insufficient information.

More studies, especially multicenter prospective randomized controlled studies with long-term follow-ups, should be conducted to compare the outcomes of the ICL and Artisan PIOLs, which are FDA-approved and CE-marked. These studies should be matched for age, degree of myopia, preoperative BSCVA, study design, and PIOL design. Further, future studies should report refractive outcomes per the proposed standards [48], [49] to make valid comparisons between studies.

Nevertheless, ICL implantation and iris-fixed PIOL implantation appear to be satisfactory refractive surgeries that have similar efficacies in correcting high myopia. ICLs are associated with better predictability, which can influence the patients' and surgeons' decision. However, surgeons should be careful in considering ICL implantation in patients with older age, more extensive myopia, smaller ACD, and shorter WTW distances [44], [50]. Selecting ICL length with a sizing algorithm, as required in a US trial [44], should be strictly obeyed due to severe complications, especially cataract and glaucoma. Additionally, previous versions of the ICL, such as V2 and V3, are not recommended.

## Supporting Information

Checklist S1 **Right-most column indicating the section (top-level heading) that contains each item according to the PRISMA checklist.**
(DOC)
